# Comparison of the Planas Functional Masticatory Angle Across Deciduous, Mixed, and Permanent Dentition Stages: A Cross-Sectional Pilot Study

**DOI:** 10.3390/dj14040213

**Published:** 2026-04-07

**Authors:** Gema Torres-Romero, Clara Guinot-Barona, Lidia Galán-López, Laura Marqués-Martínez, Esther Garcia-Miralles, Juan Ignacio Aura-Tormos

**Affiliations:** 1 Faculty of Medicine and Health Sciences, Catholic University of Valencia, San Vicente Martir, 46001 Valencia, Spain; getoro@mail.ucv.es (G.T.-R.); lidia.galan@ucv.es (L.G.-L.); laura.marques@ucv.es (L.M.-M.); 2 Department of Dentistry, Faculty of Medicine and Health Sciences, European University of Valencia, 46010 Valencia, Spain; 3 Dentistry Department, Faculty of Medicine and Dentistry, University of Valencia, 46010 Valencia, Spain; m.esther.garcia@uv.es (E.G.-M.); juan.aura@uv.es (J.I.A.-T.)

**Keywords:** Planas Functional Masticatory Angle, dentition stages, malocclusion, angle classification, orthodontics, pilot study

## Abstract

**Background**. The Planas Functional Masticatory Angle [PFMA] is a functional parameter describing mandibular trajectory during mastication. Its variation across dentition stages may reflect cross-sectional physiological functional adaptation during growth. **Methods**. A cross-sectional pilot study recruited 30 patients [10 per group: deciduous, mixed, permanent dentition] from a university dental clinic. PFMA was measured using a standardized intraoral photographic protocol, with intra-examiner reliability assessed [ICC > 0.9]. Molar relationships were classified per Angle’s classification. PFMA differences across dentition stages were analyzed using one-way ANOVA, and molar class distributions were evaluated with chi-square tests [*p* < 0.05]. **Results**. PFMA values decreased significantly from deciduous [64.7° ± 6.9] to mixed [55.5° ± 7.8] and permanent dentition [47.2° ± 9.8] [ANOVA, *p* < 0.001]. Post hoc analysis revealed significant differences between deciduous and permanent stages. No significant right–left PFMA differences were observed. Class I molar relationships predominated [70%], and no significant association was found between PFMA and molar class. **Conclusions**. This pilot study suggests PFMA decreases with dentition progression, reflecting physiological occlusal adaptation. Class I predominance supports functional symmetry, but PFMA-molar class associations require larger samples. Longitudinal studies are needed to further explore the clinical applicability of PFMA as a functional descriptor of masticatory adaptation.

## 1. Introduction

The prevalence of orthodontic treatment needs varies across populations and is influenced by multiple factors, including genetic background, oral health habits, facial morphology, and growth patterns, as evidenced by standardized oral health surveys and occlusal indices [1,2]. Malocclusion represents a major global oral health issue, with a considerable portion of individuals requiring orthodontic care to correct functional and esthetic discrepancies [1].

Occlusal stability plays a fundamental role in maintaining the functional and structural balance of the stomatognathic system, even though it is considered a dynamic rather than a static concept [3]. Stable occlusion ensures a harmonious intercuspation between the upper and lower arches, allowing proper masticatory function, speech, and force distribution across teeth. Over time, occlusal stability may change due to attrition, aging, or facial growth—making its long-term preservation a key objective in orthodontic diagnosis and treatment planning [3].

Within this context, the Planas Functional Masticatory Angle [PFMA] has been proposed as a useful parameter to assess occlusal function, mandibular dynamics, and masticatory patterns [4,5]. Planas described the PFMA as the angular trajectory of the mandible during lateral excursions, representing the minimal vertical dimension achieved during mastication. According to his concept, the side with the smaller angle corresponds to the functional or working side, reflecting greater occlusal attrition due to preferential chewing [4,5].

Importantly, PFMA should be interpreted as a dynamic functional parameter influenced by growth, dental eruption, and cumulative occlusal attrition, rather than as a direct surrogate of sagittal or skeletal malocclusion. For this reason, its variation across dentition stages may provide insight into physiological functional maturation, even in the absence of overt occlusal discrepancies [4,5].

Furthermore, the PFMA varies throughout different stages of dentition—deciduous, mixed, and permanent. This variation is attributed not only to progressive occlusal attrition from masticatory forces [5,6] but also to developmental changes such as mandibular growth, dental eruption, and neuromuscular maturation. In early dentition, minimal attrition combined with ongoing growth maintains higher angulation values, whereas in permanent dentition, cumulative attrition and functional adaptation tend to reduce this angle [5]. The PFMA thus provides a quantitative approach to evaluate occlusal efficiency, muscular symmetry, and masticatory development over time [6,7,8].

Despite its clinical potential, few studies have analyzed the evolution of the PFMA across dentition stages and its association with molar relationship patterns. Understanding these variations may contribute to better interpretation of functional asymmetries, unilateral mastication tendencies, and the establishment of occlusal balance during growth and orthodontic treatment [9,10].

Early studies have highlighted the utility of PFMA in assessing masticatory patterns in children and have established the developmental progression of dentition stages, providing a foundation for analyzing occlusal dynamics across growth phases [5,11,12].

The aim of this cross-sectional study was to explore how PFMA varies across different stages of dentition [deciduous, mixed, and permanent] and to descriptively characterize the distribution of Angle’s molar classes within the sample as an occlusal context, without presuming a functional or causal relationship between both parameters. We hypothesized that PFMA values would differ across dentition stages, reflecting functional maturation of the masticatory system, and that PFMA values would not show a significant association with Angle’s molar classification in individuals without severe occlusal discrepancies.

## 2. Materials and Methods

### 2.1. Study Design

A descriptive, observational, cross-sectional pilot study was conducted. The study protocol was approved by the Institutional Review Board [Ethics Committee] of the Universidad Católica de Valencia (Project Code: UCV/2021-2022/200, version V.1, 6 July 2022) and was performed in accordance with the ethical standards of the Declaration of Helsinki. The study aimed to evaluate variations in PFMA across different dentition stages—deciduous, mixed, and permanent—and to examine its relationship with molar class distribution.

### 2.2. Sample Size Calculation

This study was designed as a pilot study with the primary purpose of exploring PFMA behavior across dentition stages, estimating variability, and informing sample size calculation for future confirmatory studies. Therefore, a formal a priori sample size calculation was not performed.

This approach is consistent with methodological recommendations for pilot studies aimed at feasibility assessment and preliminary effect size estimation rather than formal hypothesis testing [13].

A convenience sample of 30 participants [10 per dentition stage] was considered appropriate according to methodological recommendations for pilot studies aimed at feasibility assessment and effect size estimation.

Based on the observed effect size for PFMA differences across dentition stages (η^2^ = 0.48), a future adequately powered study would require approximately 50–55 participants per group to achieve 80% power at α = 0.05.

### 2.3. Sample

A total of 30 patients were consecutively recruited from the Orthodontic Department of the Universidad Católica de Valencia UCV Dental Clinic. All participants provided informed consent prior to enrollment, and the study was conducted in accordance with the ethical principles of the Declaration of Helsinki. The sample was divided into three groups according to the stage of dentition, with 10 subjects per group: deciduous dentition [6 months to 6 years], mixed dentition (6 to 12 years), and permanent dentition [older than 12 years], on established chronologies of tooth eruption [12].

### 2.4. Eligibility Criteria

Participants were recruited among patients attending routine dental check-ups at the university dental clinic. Eligible subjects were required to be between 5 and 50 years of age, to present deciduous, mixed, or permanent dentition, and to provide written informed consent [or assent, when applicable]. Patients were excluded if they exhibited craniofacial syndromes, severe skeletal asymmetries, or a history of oral or lower limb surgery. Additional exclusion criteria included edentulism or the absence of more than six teeth, systemic or vestibular conditions that could impair mandibular movement, and recent episodes of vertigo or balance disorders within the previous month. Furthermore, patients presenting clinically evident functional occlusal interferences—such as anterior or posterior crossbite, marked occlusal discrepancies, or functional mandibular shifts during lateral excursions—were excluded.

Following the initial orthodontic assessment and record taking, all participants underwent a standardized training session to practice lateral mandibular movements under operator guidance until they were able to reproduce the movements consistently and reliably. Once the principal investigator confirmed the reproducibility of the movements for each participant, standardized photographic records were obtained. Consequently, all included participants were able to perform guided lateral mandibular movements without discomfort or deviation, allowing reliable PFMA recording.

### 2.5. Data Collection Procedures

Each participant underwent a standardized intraoral photographic protocol. All procedures were performed by the same operator to minimize inter-examiner variability.

**Figure 1:**
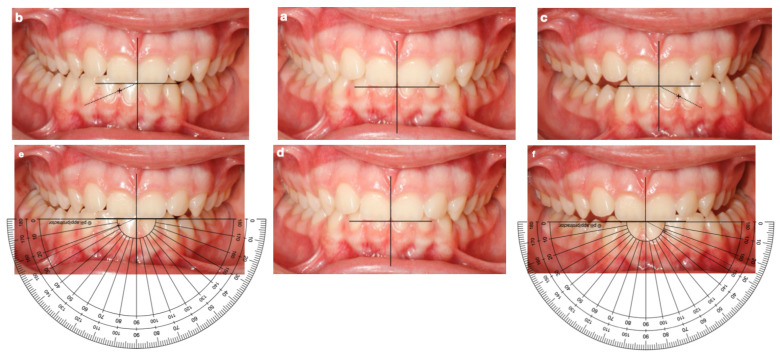
Standardized intraoral photographs obtained during mandibular movement registration focused on PFMA determination: (**a**) Maximum intercuspation [centric occlusion]. (**b**) Right lateral excursion. (**c**) Left lateral excursion. (**d**) Maximum intercuspation [centric occlusion]. (**e**) Right lateral excursion with PFMA measurement using an angular protractor (23°). (**f**) Left lateral excursion with PFMA measurement using an angular protractor (28°). According to the principles and functional laws of Planas, the observed PFMA asymmetry reflects a predominant functional tendency toward right-sided mastication in this patient.

Patients were seated in a dental chair and instructed to perform mandibular movements following verbal guidance. After placing cheek retractors, a fine-tip black marker was used to trace the upper midline on the mandibular central incisors. Three frontal intraoral photographs were taken using a Canon reflex camera (Canon Inc., Tokyo, Japan) with ring flash and an iPhone 11 Pro (Apple Inc., Cupertino, CA, USA), following this sequence: Maximum intercuspation [habitual occlusion]; right lateral excursion; left lateral excursion; and sagittal photographs of both sides (maximum intercuspation) (Figure 1).

The intraoral photographs were analyzed digitally using Adobe Photoshop 23 (2022) (Adobe Systems Inc., San Jose, CA, USA). Each image was calibrated with a known linear reference [inter-incisal distance] to correct for optical magnification and ensure measurement reliability. The PFMA was determined by drawing a horizontal line along the incisal edges of the upper central incisors and a second line following the mandibular midline trajectory during right and left lateral excursions; while this method provides a clear visual reference, its potential for slight smudging during movement is acknowledged. The angular value obtained from the intersection of both reference lines was recorded as the PFMA for each side (right and left) [4,5,11]. This photographic method for PFMA measurement has been previously validated in studies of masticatory function in different dentition stages [4].

PFMA values were expressed in angular degrees, although the parameter is interpreted as a functional descriptor rather than a diagnostic angle.

Maximum intercuspation was recorded as the habitual occlusal position. Centric relation was not specifically assessed, as PFMA evaluation focuses on functional mandibular movement rather than condylar position.

### 2.6. Intra-Examiner Reliability Assessment

To ensure measurement consistency, intra-examiner reliability was evaluated for PFMA measurements. All assessments were performed by a single calibrated examiner. A random subset comprising 10% of the intraoral photographs (3 participants; 9 images in total) was remeasured after a 2-week interval to minimize recall bias. The intraclass correlation coefficient (ICC, two-way mixed-effects model, absolute agreement) was calculated for both right and left PFMA values, yielding ICCs of 0.92 (95% CI, 0.87–0.96) and 0.94 (95% CI, 0.90–0.97), respectively—indicating excellent reliability.

### 2.7. Molar Relationship Assessment

The molar relationship was assessed according to Angle’s classification [14], in which Class I corresponds to the mesiobuccal cusp of the upper first molar occluding with the mesiobuccal groove of the lower first molar, Class II indicates a distal positioning of the lower first molar relative to the upper first molar, and Class III denotes a mesial positioning of the lower first molar. The molar class was determined and recorded independently for the right and left sides.

Canine relationships were not formally classified as an outcome variable. However, patients presenting clinically evident canine malrelationships or functional interferences involving the canine guidance that could affect lateral mandibular movements were not included in the study. All included participants exhibited a clinically stable occlusal scheme during lateral excursions, allowing reliable PFMA assessment.

### 2.8. Data Coding and Recording

All data were entered into Microsoft Excel for subsequent analysis. The recorded variables included the dentition stage [0 = deciduous, 1 = mixed, 2 = permanent], right and left PFMA] values in degrees, the mean PFMA calculated for each participant, and the molar class on both sides (1 = Class I, 2 = Class II, 3 = Class III). Data entry was double-checked to ensure accuracy and consistency prior to statistical processing.

### 2.9. Statistical Analysis

All data were coded and analyzed using SPSS v25.0 (IBM Corp., Armonk, NY, USA). Descriptive statistics [mean, median, standard deviation, and range] were calculated for all variables.

Normality was assessed using the Shapiro–Wilk test (*n* < 50). Differences in PFMA among dentition stages were analyzed using one-way ANOVA, verifying homogeneity of variances with Levene’s test. Post hoc Tukey tests were performed for pairwise comparisons.

For categorical variables [molar class distribution], frequencies and percentages were computed, and chi-square tests with Cramer’s V coefficient were applied to evaluate right–left associations. The significance threshold was set at *p* < 0.05.

Side-specific PFMA analyses were conducted to explore potential functional asymmetry between the right and left sides, which may reflect preferential or unilateral masticatory patterns. This approach allowed the identification of side-related differences in PFMA values while maintaining the overall focus on functional adaptation across dentition stages.

## 3. Results

The final sample consisted of 30 participants, with 10 subjects allocated to each of the three dentition stages. The groups were defined by age based on established tooth eruption chronologies. The mean age was 4.2 years (range: 3–6 years) for the deciduous group, 8.8 years (range: 7–11 years) for the mixed group, and 24.6 years (range: 13–42 years) for the permanent group. Due to the pilot nature and small sample size of this study, demographic characteristics such as sex were not analyzed as independent variables.

### 3.1. Descriptive Analysis

Descriptive statistics characterized Planas Functional PFMA values across dentition stages (deciduous, mixed, permanent). PFMA sums (right + left) decreased from 64.7° ± 6.89 (deciduous) to 55.5° ± 7.82 (mixed) to 47.2° ± 9.81 (permanent), with ranging from 11° (right, permanent] to 38° (left, deciduous) as outliers (Table 1).

**Figure 2:**
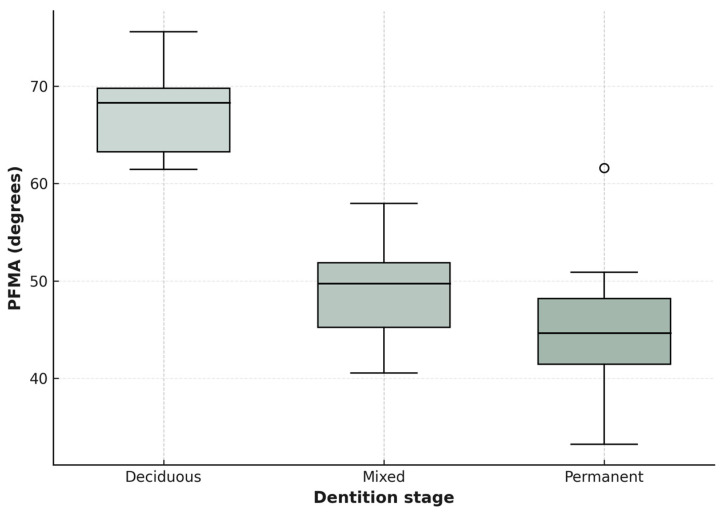
Box plot of PFMA (sum of right and left PFMA values, in degrees).

One-way ANOVA suggested significant differences in PFMA sums across groups (F(2,27) = 12.34, *p* < 0.001, η^2^ = 0.48, large effect), with post hoc Tukey tests showing significant differences between deciduous and permanent stages [*p* < 0.001], but not deciduous-mixed (*p* = 0.08) or mixed-permanent (*p* = 0.12). Shapiro–Wilk tests “suggested normality for PFMA sums and individual angles (all *p* > 0.05), and Levene’s tests verified homogeneity of variances for right (*p* = 0.756) and left PFMA (*p* = 0.062), supporting parametric analyses. Paired *t*-tests found no significant right-left PFMA differences (*p* > 0.05). These trends are visualized in Figure 2. Class I molar relationships predominated (70%, *n* = 21), followed by Class II (20%, *n* = 6) and Class III [10%, *n* = 3], with no significant association between PFMA sums and molar classes (χ^2^(4) = 3.12, *p* = 0.54).

### 3.2. Side-Specific PFMA Analysis

A complementary side-specific analysis was performed to explore whether PFMA values differed between the right and left sides across dentition stages.

Right-side PFMA values showed a significant effect of dentition stage (F(2,27) = 11.259; *p* < 0.001), with post hoc Tukey tests indicating that only the comparison between deciduous (M = 32.3; SD = 4.32) and permanent dentition (M = 22.0; SD = 5.77) reached statistical significance (mean difference = 10.3°, *p* < 0.001).

Similarly, left-side PFMA values also differed across dentition stages (F(2,27) = 6.098; *p* = 0.007), with significant differences observed between deciduous (M = 32.8; SD = 3.58) and permanent dentition (M = 25.2; SD = 6.23) (mean difference = 7.6°, *p* = 0.005).

In both analyses, Levene’s test suggested homogeneity of variances, and no other pairwise comparisons were statistically significant. Overall, these findings support a symmetrical pattern of functional adaptation without clinically relevant right–left differences.

### 3.3. Association Between PFMA and Molar Classification

A one-way ANOVA showed no significant differences in PFMA values across Angle’s molar classes (*p* = 0.642). The distribution of molar classes within the sample is reported descriptively in Table 2. Given the pilot nature of the study and the limited representation of non-Class I subjects, no further stratified analyses of PFMA by molar class were performed to avoid overinterpretation of subgroup data.

As a descriptive assessment of occlusal symmetry, a 3 × 3 contingency analysis comparing right and left molar classes showed a statistically significant association between both sides (χ^2^ = 27.18, *p* < 0.001), with a strong degree of concordance according to Cramer’s V (0.673). The bilateral Class I pattern was the most prevalent configuration, observed in approximately 60% of participants, whereas Class III combinations were the least frequent, representing about 10% of the total sample.

## 4. Discussion

### 4.1. PFMA Variation Across Dentition Stages

The findings of this pilot study indicate a progressive and statistically significant decrease in PFMA values from deciduous to permanent dentition. This pattern is consistent with a gradual process of functional adaptation of the masticatory system during growth and development. Rather than reflecting dysfunction, this reduction appears to represent a physiological adjustment mechanism, in line with the concept of functional equilibrium described by Planas [5]. Similar age-related trends have been reported in functional studies of mastication, in which cumulative occlusal attrition and neuromuscular adaptation contribute to changes in mandibular movement patterns over time [11,15].

The observed reduction in PFMA may be explained by the combined influence of progressive occlusal attrition, mandibular growth, and dental eruption. During early dentition stages, limited attrition and ongoing craniofacial development may preserve higher PFMA values, whereas in permanent dentition the establishment of a stable occlusal plane and long-term functional loading tend to be associated with lower values. From this perspective, PFMA should be interpreted as a composite functional descriptor influenced by both morphological and neuromuscular maturation rather than as an isolated structural measurement.

### 4.2. Comparison with Previous Studies

The present results are consistent with the theoretical framework proposed by Planas [5] and with previous investigations that evaluated PFMA as an indicator of masticatory patterns and occlusal function [11,15]. Studies by Latorre and Cahuana [11] and Travez Palma [4] similarly reported higher PFMA values in primary dentition and a gradual reduction across mixed and permanent dentition, supporting the association between functional maturation and changes in mandibular excursion patterns. In addition, analyses of PFMA in young children have highlighted its utility in characterizing early masticatory behavior and functional development [16], reinforcing the trends observed in the deciduous dentition group of the present study.

In growing patients, understanding the evolution of PFMA may contribute to the early identification of deviations from typical functional maturation. Previous authors have suggested that functional assessments of mastication may provide complementary information when planning interceptive or orthopedic interventions, particularly in patients with developing occlusal discrepancies [17]. However, such applications require cautious interpretation and should be supported by longitudinal and clinically comprehensive data.

### 4.3. PFMA and Molar Class Distribution

In this pilot study, no significant association was found between PFMA values and Angle’s molar classifications. This finding persisted despite the predominance of Class I relationships in the sample, a distribution that is consistent with epidemiological data reported in different populations [1,18,19,20]. These results suggest that PFMA, as a functional descriptor of mandibular trajectory, may operate independently of the sagittal molar relationship in individuals without severe occlusal discrepancies.

This lack of association should be interpreted with caution. The limited sample size, the low prevalence of Class II and III malocclusions, and the exploratory nature of the study reduce the statistical power to detect subtle relationships. In addition, the absence of clinically relevant right–left differences in PFMA values across dentition stages supports a generally symmetrical pattern of functional adaptation, consistent with reports of bilateral masticatory alternation during development [21,22]. Larger and more heterogeneous samples would be required to clarify whether specific occlusal patterns exert a measurable influence on PFMA values.

### 4.4. Clinical Implications

From a clinical perspective, the progressive reduction in PFMA values observed across dentition stages supports its interpretation as a functional descriptor of masticatory adaptation during growth, rather than as a diagnostic indicator of malocclusion. This finding is consistent with functional concepts of occlusal equilibrium and neuromuscular adaptation described in previous literature [5,7,9]. Monitoring PFMA variation may therefore provide complementary information on functional symmetry and occlusal maturation in developing dentitions [11,15]. However, given the pilot nature of the present study, PFMA assessment should be considered supportive rather than diagnostic, and its clinical applicability requires confirmation through longitudinal studies with larger and more diverse samples [23,24].

In addition, the photographic protocol used for PFMA assessment is time-efficient, low-cost, and easily reproducible in routine clinical settings, as it does not require specialized equipment or complex motion analysis systems. This practical approach may facilitate the wider implementation of PFMA evaluation in both clinical practice and educational environments.

The potential clinical benefit of PFMA assessment is expected mainly in growing patients, particularly in pediatric dentistry and orthodontics, where it may complement conventional static occlusal assessments by providing information on functional maturation and occlusal symmetry. In orthodontics, PFMA may be useful during growth monitoring or treatment follow-up, whereas in pediatric dentistry it may support early identification of atypical masticatory patterns. Its routine application in prosthodontics would be limited to selected cases and would require further validation.

### 4.5. Study Limitations

Several limitations of this pilot study should be acknowledged. First, the limited sample size [*n* = 30] restricts statistical power and generalizability. However, this should be interpreted in the context of the exploratory pilot design, which was intentionally conceived to assess feasibility and estimate preliminary effect sizes rather than to provide definitive hypothesis testing.

Second, the cross-sectional design precludes the assessment of intraindividual developmental trajectories. Accordingly, differences in PFMA observed across dentition stages reflect between-group comparisons rather than true temporal changes within the same individuals. Longitudinal studies are therefore required to confirm whether the observed patterns represent progressive functional adaptation over time.

Additionally, the wide age range within the permanent dentition group may have contributed to increased variability in PFMA values, likely reflecting heterogeneity in cumulative occlusal attrition and functional adaptation between adolescents and adults.

From an occlusal standpoint, assessment was restricted to sagittal relationships based on Angle’s molar classification, without consideration of transverse or vertical discrepancies. Future investigations should incorporate a more comprehensive occlusal evaluation, potentially supported by three-dimensional imaging techniques such as CBCT.

Finally, although the standardized photographic protocol demonstrated excellent intra-examiner reliability, more advanced motion analysis techniques could further improve measurement precision and provide additional insight into mandibular kinematics during functional movements, including three-dimensional jaw tracking systems, optoelectronic motion capture, electromagnetic mandibular tracking devices, and surface electromyography synchronized with kinematic analysis.

## 5. Conclusions

Within the limitations of this cross-sectional pilot study, PFMA values showed a progressive and statistically significant decrease from deciduous to permanent dentition, supporting its interpretation as a functional descriptor of masticatory system maturation. This developmental trend appears to reflect physiological adaptation associated with growth, occlusal attrition, and neuromuscular coordination rather than pathological change.

No significant association was observed between PFMA values and Angle’s molar classification. This finding suggests that PFMA may operate independently of sagittal molar relationships in individuals without severe occlusal discrepancies and reinforces its role as a functional rather than a morphological parameter.

Given the pilot nature of the study and the limited sample size, these findings should be interpreted cautiously. Future longitudinal studies with larger and more diverse samples, as well as more comprehensive occlusal assessments, are needed to further explore the clinical applicability of PFMA in the evaluation of masticatory development.

## Figures and Tables

**Table 1:** Descriptive statistics of PFMA values by dentition stage *.

Dentition Stage	Right PFMA Value (°)	Left PFMA Value (°)	Total PFMA (°)
Deciduous	32.3 ± 4.32	32.8 ± 3.58	64.7 ± 6.89
Mixed	27.3 ± 4.32	28.2 ± 4.52	55.5 ± 7.82
Permanent	22.0 ± 5.77	25.2 ± 6.23	47.2 ± 9.81

* Values are expressed as mean ± standard deviation (SD). PFMA values are reported in angular degrees but are interpreted as a functional descriptor rather than a diagnostic angle.

**Table 2:** Distribution of Angle’s molar classes by side (descriptive analysis).

Molar Class	Left Side *n* (%)	Right Side *n* (%)
Class I	22 (73.3%)	20 (66.7%)
Class II	5 (16.7%)	6 (20.0%)
Class III	3 (10.0%)	4 (13.3%)

## Data Availability

The data presented in this study are available on request from the corresponding author. The data are not publicly available due to ethical and privacy considerations.

## Abbreviations

The following abbreviations are used in this manuscript:

BMI	Body Mass Index
CoP	Center of Pressure
COP	Center of Pressure [alternative spelling]
COPx	Medio-lateral center of pressure displacement
COPy	Antero-posterior center of pressure displacement
EMG	Electromyography
FMA	Functional Masticatory Asymmetry
ICC	Intraclass Correlation Coefficient
PRISMA	Preferred Reporting Items for Systematic Reviews and Meta-Analyses
RoB	Risk of Bias
SD	Standard Deviation
SE	Standard Error
SR	Systematic Review
